# The Leeds Infirmary and a Justice of Assize

**Published:** 1916-08-05

**Authors:** 


### THE LEEDS INFIRMARY AND A JUSTICE OF ASSIZE.

During the hearing of a charge of manslaughter brought against a Leeds medical man, the Judge (Mr. Justice Shearman) made some strong comments upon the action of a student at the Leeds Infirmary and on the rules of the infirmary which the student was obeying. The case is of distinct importance from an institutional standpoint, and as the Judge's remarks may reach a wide audience, it is important that the issue at stake should be considered from every point of view. Probably if the judge had been fully seized of all the factors of the case he would have modified the comments which he saw fit to make.

A panel practitioner of Leeds, having presumably no connection whatever with the infirmary, and not even a Leeds-trained man, went to a confinement either drunk or drugged or both. After the birth he left the house and refused to return when requested to do so by the patient's husband. The patient soon afterwards died of hæmorrhage, due to the criminal negligence of the medical man (who has been sentenced to imprisonment). So far there is only one opinion possible as to the course of events. When the practitioner declined to return to the patient the husband went on to the infirmary and requested a student to return with him to the patient, saying that his wife was dying. The reports of the trial do not make it clear how and why this application was made to an unqualified student instead of to a practitioner or the resident obstetric officer or his (qualified) deputy. There is a rule of the infirmary that its officers may not attend a patient outside without the consent of the latter's private practitioner, if such there be. The student rang up the doctor on the telephone, and was informed that there was nothing the matter with the woman, and that he should take no notice of the husband. He acted on this advice, and shortly afterwards the woman died.

This evidence led Mr. Justice Shearman into a condemnation of the rules of the infirmary, because they prevented help being sent to a dying woman. It is easy enough to agree with him that rules of etiquette or of any other sort should not apply to prevent prompt medical aid from reaching any case of grave emergency. But as a matter of fact it was not a rule of etiquette that prevented an attempt to save this woman's life. No medical student is a fit person to treat *post-partum* hæmorrhage; and it is impossible to believe that Leeds is so bereft of medical men that not one was available; if search had been made it is practically certain that one could have been procured much more quickly than by going to the infirmary.

But, it may be anticipated, the reply will be made, "The husband may have been foolish, but having applied to the infirmary for help, surely he should have received it." To which there are two answers to be urged. One is that a voluntary general hospital does not undertake to send out its doctors to the homes of its patients, but only to supply skilled attention to those who come (or are brought) to its doors. It is true that there is an exception to this rule in respect of "district" midwifery cases: but such cases are only attended by the hospital's officers when they have been notified beforehand, and when the prospective patient has been interviewed by a qualified medical man at the hospital itself. No general hospital sends out medical attendants (qualified or unqualified) to cases of which it has no previous cognisance; nor can it do so without incurring grave responsibilities without proper means for carrying them out. The other point is that the rule is a necessary condition of hospital practice; and when a case of emergency of this sort arises outside the hospital, the proper remedy is to call in the nearest medical man without delay. The rule, in other words, is framed for the protection of the public.

But the Judge rebuked the unfortunate student very severely. It is possible, though it does not appear on the face of the newspaper reports, that the student erred in not referring the case to higher authority. But was it just to blame him for refusing to obey an emergency call when he had no sufficient evidence that an emergency existed? He had the telephonic assurance of a doctor that the patient had nothing the matter with her; he had also the rule against attending patients without the sanction of their private practitioner—a rule framed for the protection of the public. So we await the explanation of the Leeds Infirmary managers.

